# Repurposing Small Molecules to Target PPAR-γ as New Therapies for Peripheral Nerve Injuries

**DOI:** 10.3390/biom11091301

**Published:** 2021-09-01

**Authors:** Melissa L. D. Rayner, Jess Healy, James B. Phillips

**Affiliations:** 1 UCL School of Pharmacy, University College London, London WC1N 1AX, UK; jesshealy44@gmail.com (J.H.); jb.phillips@ucl.ac.uk (J.B.P.); 2 UCL Centre for Nerve Engineering, University College London, London WC1N 6BT, UK

**Keywords:** peripheral nerve injury, drug repurposing, PPAR-γ, small molecules, nerve regeneration, Rho/ROCK pathway

## Abstract

The slow rate of neuronal regeneration that follows peripheral nerve repair results in poor recovery, particularly where reinnervation of muscles is delayed, leading to atrophy and permanent loss of function. There is a clear clinical need to develop drug treatments that can accelerate nerve regeneration safely, restoring connections before the target tissues deteriorate irreversibly. The identification that the Rho/Rho-associated kinase (ROCK) pathway acts to limit neuronal growth rate is a promising advancement towards the development of drugs. Targeting Rho or ROCK directly can act to suppress the activity of this pathway; however, the pathway can also be modulated through the activation of upstream receptors; one of particular interest being peroxisome proliferator-activated receptor gamma (PPAR-γ). The connection between the PPAR-γ receptor and the Rho/ROCK pathway is the suppression of the conversion of inactive guanosine diphosphate (GDP)-Rho to active guanosine triphosphate GTP-Rho, resulting in the suppression of Rho/ROCK activity. PPAR-γ is known for its role in cellular metabolism that leads to cell growth and differentiation. However, more recently there has been a growing interest in targeting PPAR-γ in peripheral nerve injury (PNI). The localisation and expression of PPAR-γ in neural cells following a PNI has been reported and further in vitro and in vivo studies have shown that delivering PPAR-γ agonists following injury promotes nerve regeneration, leading to improvements in functional recovery. This review explores the potential of repurposing PPAR-γ agonists to treat PNI and their prospective translation to the clinic.

## 1. Introduction

Nerve damage resulting from a peripheral nerve injury (PNI) can be highly debilitating to a patient causing life-long loss or disturbance to end-organ function [1]. The most common cause of such injuries is trauma as a consequence of motor vehicle accidents for example [2]. However, injuries can also be caused by surgery, chemotherapy, radiation therapy in breast, head and neck cancers, and birth complications [3].

Despite advancements made in PNI research, effective treatment acting to improve functional recovery remains challenging [4]. The current treatment for PNI is surgical intervention, with a primary repair between the proximal and distal stump being the gold standard therapy for transection injuries with no tension, and microsurgical autografts for long gap repair [5]. However, they do not address the complexity of cellular and molecular events occurring along the entire length of the nerve [5] and adequate functional improvement is not always achieved, therefore there is a clear clinical need to find new therapeutic approaches.

Drug treatments that promote axonal regeneration with correlating functional recovery could provide an alternative treatment. Current drug therapies tend to focus on the resulting symptoms from a PNI such as neuropathic pain, inflammation, and weakness without modifying the condition itself [6]. Appropriate drug agents would need to target specific events following a nerve injury, for example to maintain neuronal viability, encourage axonal growth over long distances, and improve axonal specificity to end-organ targets [7].

Unlike the central nervous system (CNS) the peripheral nervous system (PNS) does have some innate regenerative capacity, however, the rate of this regeneration is remarkably slow (~1 mm/day) [8,9]. This delay increases the likelihood of Schwann cell degeneration or senescence in denervated distal nerve segments leading to a loss of their supportive role towards the regenerating axon and consequently poor functional outcomes [10,11]. This is mainly due to the decreased production of the neurotrophic factors, cytokines, and axon adhesion molecules produced by these cells, which are needed to support the intrinsic growth capacity of neurons and provide a permissive environment [12]. Successful regeneration is highly dependent on both efficient axonal regrowth and myelination of regenerated axons by Schwann cells [12,13]. Furthermore, a lack of connection between the axons and target organ results in atrophy of the target organ and the associated loss of functional recovery [4,8,14,15]. Increasing the rate of regeneration through the use of drug therapies could meet a clear clinical need and provide an effective novel treatment strategy for PNI.

This review explores how the Rho/ROCK pathway could be modulated in PNI, in particular via the peroxisome proliferator-activated receptor gamma (PPAR-γ). Articles that have studied the use of drugs or small molecules that target PPAR-γ are reviewed, with a particular focus on where these have been used to promote regeneration or functional recovery following PNI. The 86 articles included were published between 1995 and 2021. Articles that studied PPAR-γ in optic nerve, neuropathic pain, neuroprotection, inflammation or neurodegenerative disease were excluded. The study of PPAR-γ in other nervous system disorders is important; however, this review focuses specifically on peripheral nerve regeneration and functional recovery.

## 2. Signaling Pathways Involved in PNI

**Figure 1:**
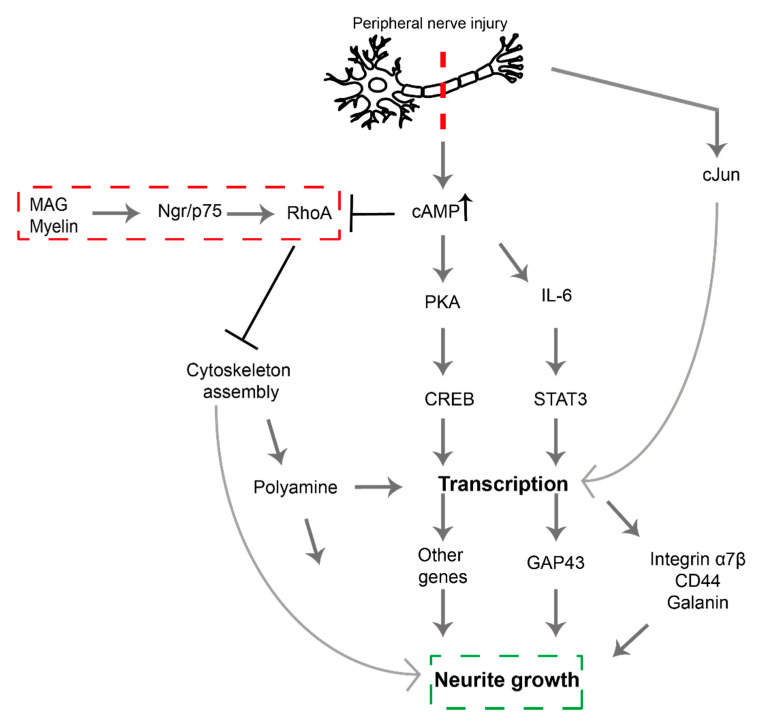
Signaling pathways following peripheral nerve injury. All pathways are excitatory acting to enhance axonal growth except the Rho/ROCK inhibitory pathway indicated in red. Modified from [12]. Myelin-associated glycoprotein (MAG), Nogo receptor (Ngr), p75 neurotropic receptor (p75 ^NTR^), Ras homolog family member A (RhoA), Cyclic Adenosine Monophosphate (cAMP), Protein kinase A (PKA), cAMP-response Element-Binding Protein (CREB), Interleukin-6 (IL-6), Signal Transducer and Activator of Transcription 3 (STAT3) [19].

Studies have demonstrated the effect of signaling pathway modulation on nerve regeneration (Figure 1) [16,17]. Previous studies have focused on excitatory pathways that act to enhance regeneration [18], however, one inhibitory pathway, the Rho/ROCK pathway, has been identified which could also provide targets for the development of therapeutics (Figure 1). Activation of this pathway leads to phosphorylation of downstream effector proteins—such as myosin light chain (MLC), LIM kinase (LIMK), and collapsin response mediator protein 2 (CRMP2)—and ultimately leads to growth cone collapse. Following an injury, transcription-dependent processes such as the elevation of cyclic adenosine monophosphate (cAMP) levels, act to oppose this pathway thus supporting regeneration [18].

**Figure 2:**
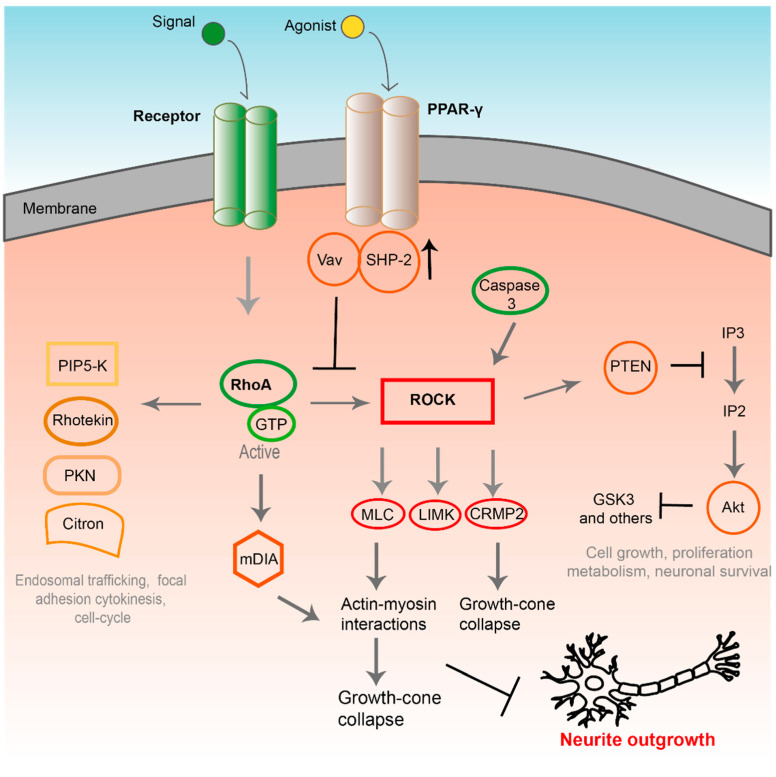
PPAR-γ receptor and its effect on downstream pathways that are potential targets for drug agents. Including the Rho/ROCK inhibitory pathway which when blocked stops growth-cone collapse and encourages neurite outgrowth. Proliferator-activated receptor gamma (PPAR-γ), Src homology region 2–containing protein tyrosine phosphatase-2 (SHP-2), Ras homolog family member A (RhoA), guanosin-5′-triphosphate (GTP), phosphatidylinositol 4-phosphate-5 kinase (PIP5K), protein kinase N (PKN), Rho-associated kinase (ROCK), myosin light chain (MLC), LIM kinase (LIMK) and collapsin response mediator protein 2 (CRMP2), phosphatase and tensin homolog (PTEN), inositol triphosphate (IP3), inositol biphosphate (IP2), serine/threonine protein kinase B (Akt) [19].

In brief, activation of the GTPase Rho (or RhoA) to its GTP-bound form with the help of downstream effector kinase ROCK leads to stiffening of the actin cytoskeleton, this initialises changes in the signaling of many downstream effectors, which in turn inhibits axonal elongation and mediates growth cone collapse (Figure 2) [16,20,21]. This underlines the potential usefulness of developing treatments that act directly upon Rho or ROCK.

Rho, together with Rac and Cdc42, belongs to the family of small GTPases, which have a role in cellular motility and cytokinesis [22,23]. Modulation of Rho, Rac1, and Cdc42 GTPase activity has been demonstrated to affect various aspects of dendritic development and influences neuronal information processing [24]. Evidence has accumulated to support the essential role played by Rho GTPases in nerve cell function and survival and axonal growth through the orchestration of growth cones [25]. One of the primary outcomes of Rho activation, and of particular relevance here, is the reorganisation of the actin cytoskeleton (Figure 2) [23,26,27,28]. As the cytoskeleton stiffens this induces downstream effectors, which inhibits axonal elongation and mediates growth cone collapse thus inhibiting nerve regeneration [16,20,21].

Rho GTPases can be viewed as molecular switches whose activity depends upon regulatory proteins specific to each family member; these include guanine nucleotide exchange factors (GEFs), GTPase activating proteins (GAPs), and guanine nucleotide dissociation inhibitors (GDIs). Inhibitors targeting these proteins would provide a basis to control neuronal growth following PNI, however, a lack of specific tool compounds has hampered progress [23,29]. Rho GTPases have long been considered ‘undruggable’ due to a combination of factors; (1) the micro molar GTP concentration found in cells and (2) lack of additional tractable binding sites [30]. Increased understanding of the structural basis for the interaction between Rho and its regulatory proteins may help to plug this gap, and some progress has been made towards this goal.

The Rho-associated kinase (ROCK) is the most extensively studied downstream effector in the Rho pathway. ROCK is a serine/threonine protein kinase belonging to the AGC family [23,31]. Two isoforms of ROCK exist and are characterised by their spatially differential expression; ROCK I is found in non-neuronal tissues and ROCK II is predominantly in the brain and muscle tissues [22,23,28]. Activation of ROCK leads to phosphorylation of downstream proteins such as MLC, LIMK, and CRMP2 leading to stiffening of the cytoskeleton and growth cone collapse. This kinase target has been extensively studied and a number of inhibitors have been reported.

The Rho/ROCK pathway (Figure 2) can also be modulated through activation of upstream receptors, such as the receptor tyrosine kinases; Eph, G-protein coupled receptors, plexins, ApoER2 [32] and the PPAR-γ [33], and through the action of neurogenic inhibitors such as MAG, Nogo-A, and CSPGs (chondroitin sulphate proteoglycans). These inhibitory signals appear to converge on the Rho GTPase pathway ultimately blocking effective regeneration [18,29] and provide additional targets for the inhibition of this signaling pathway. As the Rho/ROCK signaling pathway appears to act as a nexus for opposing nerve regeneration it has attracted considerable interest and several potential targets for drug therapies have been identified. The success of such treatments will be dependent on their ability to inhibit the downstream activity of Rho on actin cytoskeleton remodelling in the growth cones which is essential for axonal regrowth [29,34].

Studies have clearly demonstrated that neurons, particularly motor neurons, have an increased responsiveness to the Rho/ROCK pathway following PNI [18,34] further highlighting the potential of this pathway as a target for pharmaceutical intervention. Blocking this pathway, which prevents growth cone collapse, has shown promising results both in vitro and in vivo [18,29,34,35,36,37,38].

## 3. Proliferator-Activated Receptor Gamma (PPAR-γ) Activation

PPAR-γ is a member of the nuclear receptor family that heterodimerizes with retinoic acid-X receptors and is rendered transcriptionally active by binding to a specific DNA sequence element termed the PPAR response element [33,39]. Activation of PPAR-γ subsequently leads to inhibition of the Rho/ROCK pathway via upregulation of the protein tyrosine phosphatase, Src homology region 2–containing protein tyrosine phosphatase-2 (SHP-2). This cytosolic protein tyrosine phosphatase (PTP) dephosphorylates the Rho-GEF Vav. This inactivation suppresses the conversion of inactive GDP-Rho to active GTP-Rho, ultimately resulting in the suppression of Rho/ROCK activity [33]. The development of potent selective and cell permeable inhibitors of PTP’s is challenging due to the conserved and highly polar nature of the active site coupled with the fact that anionic phosphate is the key recognition motif [40]. The connection between PPAR-γ and the Rho/ROCK pathway is supported by an in vivo study in adult rats, which demonstrated suppression of Rho/ROCK activity was consistent with upregulated SHP-2 expression and inactivation of Vav following 4 weeks treatment with the PPAR-γ agonist, pioglitazone [33].

More specifically, ligands bind to the ligand-binding pocket (LBP) of PPAR-γ modulating the activation function 2 (AF-2). AF-2 is a coactivator binding surface comprised of residues from the C-terminal helices (H) 12, H3 and H5 [41,42]. Agonist binding results in increased conformational rigidity of H12, locking the protein into an active conformation, which enables co-activator binding (via the conserved LXXLL motif) and results in formation of a transcriptionally active form of the receptor. The binding mode of the respective ligands and the associated extent of stabilisation of H12 in the active conformation correlates with the magnitude of the transcriptional response. Full agonists, for example, occupy both sub-pockets of the Y-shaped LBP, forming strong contacts with a tyrosine residue (Tyr473) on the inner surface of H12. This results in H12 maintaining a rigid active position, promoting AF-2 activity [41,43]. Partial agonists, on the other hand, can adopt multiple conformations, with multiple copies of the ligand occupying 1 or both sub pockets, and may or may not directly interact with key residue Tyr473.

## 4. PPAR-γ in Peripheral Nerves

The role of PPAR-γ in nerve tissue remains unclear, however, immunohistochemical analysis has demonstrated that PPAR-γ can be found in Schwann cells of myelinated fibres and endothelial cells in rat peripheral nerves [44]. This was reinforced by another study in which immunofluorescence staining demonstrated the expression of PPAR-γ within Schwann cells of healthy and regenerating nerves 1 week following a crush injury [45]. PPAR-γ was found to have a role in the inflammatory process in Schwann cells [46].

A recent study has suggested that PPAR-γ activation has a role in controlling the phenotype of Schwann cells [47]. Following an injury the Schwann cells reprogram to a repair state to aid regeneration and only in a later post-injury phase switch back to their myelinating state [48]. This repair state was shown to coincide with changes in Schwann cell lipid metabolism. Inhibition of S1P/PPAR-γ stalled lipid production and induced Schwann cell repair phenotype. Pharmacologically up-regulation of PPAR-γ activity enhanced lipogenic genes, while PPARγ inactivation decreased their expression. Therefore, S1P/PPAR-γ inhibition appears necessary for initiating the Schwann cell switch towards a repair state after injury. Application of PPAR-γ agonist might therefore be beneficial during later regeneration stages for the final re-myelinating phase [47]. Moreover, the presence of PPAR-γ has been confirmed in axons within a rat sciatic nerve model in which PPAR-γ activation increased at 2, 4, and 6 h after a nerve ligation or crush injury [49]. Another study found PPAR-γ was also expressed in human neuroblastoma cells and identified a correlation between expression and the maturational stage of the cell, thus indicating that PPAR-γ has a role in nerve cell biology [50], including the development and health of neurons [39].

Treatment with the PPAR-γ agonist, troglitazone in a primary rat hippocampal culture induced neurite outgrowth and increased axon length [51]. This was echoed in another study in which rosiglitazone increased the activation of PPAR-γ in rat primary cortical neurons resulting in increased neurite outgrowth. The opposite was seen when treating the neurons with the PPAR-γ antagonist, GW9662 [49].

PPAR-γ ligands can also be beneficial to improve neuro-inflammation and treat neuropathic pain [39,52]. PPAR-γ exerts its anti-inflammatory and neuroprotective effects through NFkB, AP-1, STATs, and iNOS [53,54]. Over the last two decades studies have shown that cannabinoid compounds interact with PPAR receptors [55] and have demonstrated their ability to reduce neuroinflammation and neurodegeneration in cell and animal models [54,56,57].

Further studies have indicated that PPAR-γ activation has beneficial effects against oxidative stress, mitochondrial dysfunction, and apoptosis in several cell-based models for degenerative diseases such as Alzheimer’s disease, Huntington’s disease, amyotrophic lateral sclerosis, and spinal cord injury [58,59]. PPAR-γ activation in mouse models of the diseases listed above resulted in decreased cognitive decline [39]. Finally, evidence suggests that PPAR-γ has an effect on neuronal differentiation through influencing transcription and the activation of secondary pathways impacting cell morphology and protein expression [45]. It is evident that there are many possible benefits for the development of PPAR-γ agonists for the treatment of PNI.

## 5. Repurposing Drugs and Small Molecules to Target PPAR-γ in PNS

Drug discovery and development is expensive and time-consuming with a high risk of failure, therefore more companies and academic researchers are exploring drug repurposing as an alternative [60]. This technique employs data mining, bioinformatics and screening platforms to identify drugs that are already used in other clinical indications and reposition them for new applications [60]. Drugs with potential to promote nerve regeneration have been identified as a result of this repurposing approach and in particular, agents targeting PPAR-γ currently used for the treatment of other clinical conditions have already shown benefit in preclinical nerve regeneration studies (Table 1).

Agonists for the PPAR-γ usually contain a lipophilic backbone and an acidic moiety, usually a carboxylate [41] and many non-steroidal anti-inflammatory drugs (NSAIDs) such as ibuprofen (Table 1) fulfil these structural characteristics and have demonstrated different degrees of partial agonism for PPAR-γ [41].

To date promising results have been reported for treatment with ibuprofen and diclofenac treatment following PNI, for example Madura et al., 2011 demonstrated an improvement in functional recovery and remyelination of axons after 3 months following 3 weeks ibuprofen treatment in a rat tibial nerve transection model. Furthermore, treatment with diclofenac delivered to a transection of a rat sciatic nerve in an artery graft elicited improvement in functional recovery and axon regeneration [64].

To the best of our knowledge other NSAIDs have yet to be investigated in PNI models; however, the relative affinities of a number of NSAIDs for PPAR-γ have been determined [41] and follow the rank order sulindac sulfide > diclofenac > indomethacin > ibuprofen. Given the promising results obtained with ibuprofen and diclofenac, further investigation of NSAIDs as agents to promote regeneration may prove fruitful. It is likely that greater understanding of how NSAIDs interact with PPAR-γ will help the development of more effective drugs targeting this receptor following PNI [49].

Another class of drugs that act upon PPAR-γ is the glitazones which are more commonly used as anti-diabetics. Pioglitazone and rosiglitazone have, however, been studied for use in PNI. In a mouse sciatic nerve crush injury model, three weeks of treatment with pioglitazone improved myelination [65], further supporting the proposal of PPAR-γ as a promising target for peripheral nerve regeneration.

NSAIDs have also demonstrated positive effects on regeneration in the CNS by inhibiting Rho activation [68]. In both the PNS and CNS, however, the mechanisms appear independent of their pharmacological activity as cyclooxygenase inhibitors [68,69]. Of the NSAIDs ibuprofen has been the most extensively studied and has been demonstrated to inhibit the Rho signaling pathway more selectively than the other NSAIDs such as indomethacin [68]. Both ibuprofen and indomethacin demonstrated positive effects on neurite elongation in cell culture models [61,70]. This has been extrapolated into animal models with positive effects demonstrated on neurite elongation [62,63], and improvements seen in myelination and functional recovery [69,71,72] with ibuprofen.

Duan et al. reported that three days of treatment in vitro with ROCK inhibitors Y27632 and fasudil lead to tolerance of ROCK inhibition and attenuation of neurite outgrowth, but the tolerance was decreased with the cyclooxygenase-2 (COX-2) inhibitor NS398 [73]. This tolerance appeared to correlate with upregulation of the COX-2 pathway. The authors demonstrated a synergistic effect on inhibition of both ROCK and COX-2, suggesting that dual treatment may have promise [73]. Further work is required to validate this observation in vivo, but it may have important implications for the evaluation of novel therapies for nerve regeneration.

A preclinical study combining the treatment of the ROCK inhibitor fasudil and COX-2 inhibitor celecoxib demonstrated a synergistic effect by decreasing COX-2 and ROCK-II activation in a spinal cord injury site following a hemisection at the crest of T_11_ in rats [74]. The locomotor functional recovery was enhanced with combined therapy but not when the two drugs were given alone, suggesting again that dual treatment may have promise [74].

To address this hypothesis another study was conducted using the anti-inflammatory drug methylprednisolone and the PPAR-γ agonist rosiglitazone, which also demonstrated a positive synergistic effect. The combined treatment of methylprednisolone and rosiglitazone following a laminectomy and compressive spinal cord injury in rats, had a pronounced effect on attenuation of inflammation and apoptosis. Increased functional recovery was observed when the two drugs were administered together in comparison to using either drug treatment alone [75].

## 6. PPAR-γ in the Central Nervous System

This review has focused on targeting PPAR-γ in the PNS, however, there are studies that have explored targeting PPAR-γ in the CNS [76,77,78,79]. An evaluation of the published literature revealed that an extensive list of drugs or small molecules targeting PPAR-γ have been tested in injuries of the CNS (Table 2).

One recent study that is out of the inclusion criteria of this review but could have an influence on the study of PPAR-γ for CNS or PNS treatments is a randomised, placebo- controlled trial that found no correlation between ibuprofen dose and PPAR-γ gene expression during an emotion-related neural activation [85].

## 7. Conclusions and Future Work

To conclude, there is evidence in support of targeting PPAR-γ in peripheral nerve regeneration, which provides a platform for the development of pharmacological interventions. The repurposing of approved drugs is likely to be valuable in moving therapies rapidly towards the clinic. Although there have been many advancements made in the identification of targets for pharmacological agents to treat nerve injury, the field is still in its infancy. Only a few agents have been directly tested in humans to date for applications related to peripheral nerve function. Ibuprofen as well as other NSAIDs are routinely used for inflammation and pain and already have well-established safety and efficacy profiles [20]. Therefore, it would be feasible to test such drugs in humans following nerve injury with a low risk of toxic effects. Furthermore, the need for extensive pre-clinical studies can be a drawback in the development of new drugs for treating nerve injuries [20], making the repurposing of established drugs an attractive alternative proposition.

Future work would need to address the additional issues associated with method and site of drug administration [20] to minimise problems associated with toxicity, off-target effects and efficacy. Recent studies have suggested the use of conduits to deliver drugs locally to the site of injury may overcome these problems [20], providing an exciting alternative to systemic administration.

## Figures and Tables

**Table 1:** Peripheral nerve studies that have explored the regenerative or functional recovery effects of drugs and small molecules that target PPAR-γ.

Compound	Chemical Structure	Model	Effect on Nerve Regeneration	Reference
Ibuprofen	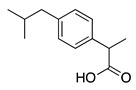	In vivo: Interpositional graft on adult rat tibial nerve; treated through osmotic pumps. In vitro: NG108-15, DRG and 3D co-culture. In vivo: Transection with primary repair in sciatic nerve treated through osmotic pump. In vivo: Transection with primary repair or crush injury in sciatic nerve treated through biomaterials.	Recovery of TFI and increase of area of axon and myelin. In vitro: Elongation of neurites In vivo: Increase in axon number. Increase in axon number and functional recovery.	[61,62,63]
Diclofenac	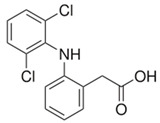	In vivo: Sciatic nerve transection with artery graft filled with diclofenac.	Improved functional recovery and faster recovery of regenerated axons.	[64]
Sulindac sulfide	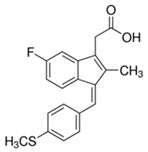	In vivo: Transection with primary repair or crush injury in sciatic nerve treated through biomaterials.	Improved functional recovery.	[63]
Pioglitazone	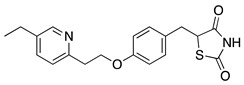	In vivo: Crush injury on sciatic nerve in CD36-deficient mice. In vivo: Bilateral cavernosal nerve crush injury.	Improved re-myelination. Protective effect on pelvic ganglion neurons.	[65,66]
Rosiglitazone	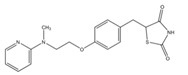	In vitro: N2A cell culture.	Promoted neurite outgrowth and increased population of neurite-bearing cells.	[67]

This includes approved drugs as well as small molecules in experimental stages. Inclusion criteria selected literature that had studied PPAR-γ as a target to block or activate the Rho/ROCK pathway and no other signaling pathways. Drugs or small molecules targeting CNS, optic nerve, neuropathic pain, inflammation, or neurodegenerative diseases were also excluded. Relevant articles were identified and obtained from PubMed up until 4 August 2021.

**Table 2:** Studies that have explored the effect of drugs and other experimental small molecules targeting the PPAR-γ in the CNS.

Compound	Clinical Indication	Reference
Ibuprofen	CNS injury	[70]
	CNS injury	[61]
	Spinal cord injury	[69]
	Spinal cord injury	[72]
	CNS injury	[61]
Indomethacin	CNS injury	[61]
	Spinal cord injury	[72]
Rosiglitazone	CNS injury	[49]
	Spinal cord injury	[80]
	Cerebral Ischemia injury	[81]
	Spinal cord injury	[82]
Pioglitazone	Spinal cord injury	[80]
	Spinal cord injury	[83]
Mifepristone	Cerebral ischemia-reperfusion	[84]
	injury	

Drugs or small molecules targeting neuropathic pain, inflammation or neurodegenerative diseases were excluded. Relevant articles were identified and obtained from PubMed up until 4 August 2021.

## Abbreviations

3D	3-dimensional
AF2	Activation function 2
Akt	Serine/threonine protein kinase B
AP-1	Activator protein 1
cAMP	Cyclic adenosine monophosphate
CNS	Central nervous system
COX-2	Cyclooxygenase-2
CREB	cAMP-response Element-Binding Protein
CRMP2	Collapsin response mediator protein 2
CSPG	Chondroitin sulphate proteoglycans
GAP	GTPase activating proteins
GDI	Guanine nucleotide dissociation inhibitors
GDP	Guanosine diphosphate
GEF	Guanine nucleotide exchange factor
GTP	Guanosine triphosphate
H	Helix
IL-6	Interleukin-6
iNOS	Inducible nitric oxide synthase
IP2	Inositol biphosphate
IP3	Inositol triphosphate
LIMK	LIM kinase
MAG	Myelin-associated glycoprotein
MLC	Myosin light chain
NFkB	Nuclear Factor kappa-light-chain-enhancer of activated B cells
Ngr	Nogo receptor
NSAIDs	Non-steroidal anti-inflammatory drugs
p75 ^NTR^	p75 neurotropic receptor
PIPK5	Phosphatidylinositol 4-Phosphate-5 kinase
PKA	Protein kinase A
PKN	Protein kinase N
PNI	Peripheral nerve injury
PNS	Peripheral nervous system
PPAR-γ	Peroxisome proliferator-activated receptor gamma
PTEN	Phosphatase and tensin homolog
PTP	Protein tyrosine phosphatase
RhoA	Ras homolog family member A
ROCK	Rho-associated kinase
SHP-2	Src homology region 2–containing protein tyrosine phosphatase-2
STAT3	Signal transducer and activator of transcription 3
TFI	Tibial functional index
Tyr473	Tyrosine residue 473
